# Studying the relationship between mental health literacy and emotional state among young people in Republic of Bashkortostan

**DOI:** 10.1192/j.eurpsy.2022.1778

**Published:** 2022-09-01

**Authors:** S. Galyautdinova, M. Khukhrin, T. Chuikova

**Affiliations:** 1 Bashkir State University, Department Of Psychology, Ufa, Russian Federation; 2 Bashkir State Pedagogical University, Department Of Psychology, Ufa, Russian Federation

**Keywords:** mental health literacy, young people, emotional state, Republic of Bashkortostan

## Abstract

**Introduction:**

Mental health literacy (MHL) could be defined as a knowledge of mental health, comprehension of its importance, adequate attitude to information about mental health, possession of skills and abilities to provide assistance and self-help to maintain and promote mental health.

**Objectives:**

The aim of the present study is to identify possible relationships between the components of mental health literacy and the manifestations of the emotional sphere of the respondents.

**Methods:**

The sample consisted of 220 young people from 16 to 23. Emotional Empathy Questionnaire (A. Mehrabian, M. Epstein), self-assessment of mental states (G. Eysenck), questionnaire from the study of Reavley et al., and the custom MHL questionnaire were used.

**Results:**

Most significant of relationships revealed by Pearson’s Product-moment correlation analysis: Adequate actions of respondents in a mental health-threatening situation inversely correlates with anxiety (r=-0.38, p=0.0002), aggressiveness (r=-0.22, p=0.017), frustration (r=-0.35, p=0.00008), rigidity (r=-0.29, p=0.0012). Comprehension of the importance of specialized care inversely correlates with frustration (r=-0.2, p=0.027). Helping others in difficult situations correlates with empathy (r=0.48, p=0.0000).

**Conclusions:**

A low level of the behavioral component of MHL is often manifested with high anxiety, aggressiveness, rigidity, frustration. Correlation between the cognitive component and frustration may indicate that sufficient level of this component does not allow the individual to fall into a state of deep frustration in mental health-threatening situations. Individuals with low level of anxiety, rigidity, aggression are more inclined to understand the importance of seeking professional help. An individual with lower frustration and aggressiveness will less likely choose destructive behaviors (addictions to alcohol, self-harm, etc.).

**Disclosure:**

No significant relationships.

